# Assessment of Periodontal status in Patients with Psoriatic Arthritis: A retrospective, case-control study

**DOI:** 10.4317/jced.58125

**Published:** 2021-08-01

**Authors:** Supriya Mishra, Lynn Johnson, Sangita Agrawal, Shikha Rajput

**Affiliations:** 1 MDS, Resident, Department of Periodontics, Government Dental College, Raipur, Chhattisgarh state, India; 2 MDS, Lecturer, Department of Periodontics, Maitri College of Dentistry and Research Centre, Anjora, Durg, Chhattisgarh state, India; 3 MDS, Reader, Department of Periodontics, Government Dental College, Raipur, Chhattisgarh State, India; 4 MDS, Senior Lecturer, Department of Periodontics, Mansarovar Dental College, Bhopal, Madhya Pradesh

## Abstract

**Background:**

Psoriatic arthritis (PsA) and periodontitis both represent chronic inflammatory disorders that share similar pathophysiological processes. However, very few studies have been done to address the link between the two diseases which remains poorly understood. The present study aimed to assess and compare the periodontal status in patients suffering from PsA and systemically healthy subjects to identify whether a possible association exists between PsA and periodontitis.

**Material and Methods:**

Periodontal parameters – PI, BOP, mGI, PPD and CAL were recorded in 110 patients with PsA and 110 age- and gender-matched systemically healthy patients. Mean values of the periodontal parameters were calculated for both groups and subjected to statistical analysis. Logistic regression analysis was performed to correlate the demographic data with periodontitis.

**Results:**

The frequency of periodontitis and mean values of BOP, mGI, PPD and CAL were found to be significantly higher in patients with PsA than in systemically healthy controls. The number of patients with stage III periodontitis was found to be significantly higher in the PsA group.

**Conclusions:**

A possible link exists between periodontitis and psoriatic arthritis, as exhibited by the results of the present study. Dental and medical health professionals should be aware of this relationship depending on which, they should carry out adequate treatment strategies involving periodic periodontal evaluation and care.

** Key words:**Periodontitis, psoriatic arthritis, chronic inflammation, probing pocket depth, clinical attachment loss.

## Introduction

Psoriatic arthritis (PsA) is described as chronic inflammatory arthropathy associated with psoriasis, in which the rheumatoid factor usually appears as seronegative (1). Approximately 20% of psoriatic patients can develop PsA (2,3) with the involvement of the skin, nails, entheses, bones, tendons, ligaments, synovial membrane and joints (4,5). Despite the evident role of genetic factors and autoimmune dysfunction in the basic pathophysiology, a complex interplay between immunologic and inflammatory events in PsA is also a significant component, a closer look into which reveals that there is a prominent lymphocytic invasion of skin, joints and inflamed entheses replete with activated T-cells and T-cell-derived cytokines, including interleukin (IL)-1, IL-2, IL-10, IL-17, IL-23, interferon (IFN) and tumour necrosis factor (TNF-α) (6). TNF-α enhances the production of matrix metalloproteinases (MMPs), which play a crucial role in cartilage degradation6. Besides, the over-expression of TNF-α within the joint may also result in abnormal bone remodelling (6). Furthermore, as RANKL and IL-1 are activated in the subchondral bone, TNF-α_stimulated monocytes differentiate into osteoclast precursor cells and mature osteoclasts (7).

Periodontitis is a chronic inflammatory response that occurs as a result of predominantly gram-negative anaerobic or aerobic bacterial infection originating from dental plaque (8). It is a prototype of low-grade local infection associated with a moderate systemic inflammatory response (9) and is characterized by immunologically mediated destruction of periodontal tissues, resulting in periodontal pocket formation, progressive attachment loss, marginal alveolar bone resorption, and/or gingival recession (10).

It is hypothesized that both PsA and periodontitis are immune-mediated inflammatory diseases and appear to share similar pathophysiology that includes an excess accumulation of pro-inflammatory cytokines in various parts of the body like skin, joints and periodontal tissues, a heightened immune response to the microbiota inhabiting the epithelial surface of skin, or periodontal tissues, bone/cartilage resorption and their abnormal remodelling (10). Moreover, PsA has been strongly linked to several comorbidities like coronary heart disease, metabolic syndrome, obesity, diabetes mellitus, dyslipidemia and osteoporosis (11). Similarly, periodontitis has also been associated with co-morbidities like diabetes, atherosclerosis, and stroke, which further suggests common pathways in the pathogenesis of PsA and periodontitis (12-14). Furthermore, a study has reported higher concentrations of oral bacterial DNA (**P. gingivalis**, *T. forsythia* and *Treponema species*) in the synovial fluid of patients with PsA, suggesting a perpetual involvement of periodontal pathogens in PsA (15).

Extensive research has been done on the relationship between periodontitis and systemic diseases. In the recent years, several studies have focussed on the relationship between periodontitis and psoriasis and have suggested a positive association between the two diseases (16-18). PsA is considered as a major co-morbidity of psoriasis that shares a common pathogenetic mechanism and significantly affects an individual’s quality of life. It also shares a similar patho-physiological link with periodontitis just like psoriasis; however, the disease is often overlooked. As a result, there is a dearth of evidence that has addressed the possible association between PsA and periodontitis, with questionable results (19). Hence, the current study aimed to assess and compare the periodontal status in patients suffering from PsA and systemically healthy subjects to identify whether a possible relationship exists between PsA and periodontitis. The study also attempted to analyze the extent to which various confounders affect the association.

## Material and Methods

The current study, with its case-control experimental design, comprised a total of 220- age and gender-matched participants. Considering the estimated prevalence of newly diagnosed cases of PsA patients to be 7.22% as per the results reported by Kumar *et al*. (20), the sample size required for the present study was 106 for the PsA group at 95% confidence level and absolute precision of 5%. This was rounded off to 110 in the case of drop out during the examination process. Hence, Group I included 110 newly diagnosed PsA patients not under treatment for the disease, between the age group 25-50 years and satisfying the CASPAR classification criteria of PsA, while group II consisted of 110 systemically healthy controls. Data collection was done during a period of eighteen months, starting from April 2016 to September 2017. Newly diagnosed PsA patients were randomly enrolled from the Department of Rheumatology, Apollo BSR Hospital, Bhilai, India, while the systemically healthy controls were enrolled randomly from the Department of Periodontology, Maitri College of Dentistry and Research Centre (MCDRC), Bhilai, India. The research protocol was approved by the Institutional Ethical Review Board of MCDRC, Bhilai, India and written informed consent was obtained from all the participants.

Subjects with a history of any known systemic disease (other than PsA for group I) or any inflammatory disorders, use of tobacco in any form, pregnant women and lactating mothers, those under any medication and those undergone periodontal therapy in the last 6 months prior to the examination, were excluded from group I and group II.

-Periodontal examination

A single, trained examiner and blinded about the study design (LJ) assessed both the groups for periodontal status. Clinical parameters recorded for both the groups were – plaque index (PI), Bleeding on Probing (BOP), modified gingival index (mGI), probing pocket depth (PPD) and clinical attachment level (CAL). The intraoral examination was done using a mouth mirror and a graduated periodontal probe and the measurements were rounded off to the nearest millimetre. PI and mGI were recorded in all the participants for the entire dentition, excluding the third molars at four points namely – mesiobuccal papilla, buccal margin, distolingual papilla and the entire lingual margin as per the criteria given by Loe and Sillness (21) and Lobene *et al*. (22) respectively. BOP was recorded as presence or absence of gingival bleeding within 30 seconds of probing through the gingival sulcus of buccal and lingual surfaces of all teeth except third molars. It was computed as the ratio of number of sites with gingival bleeding to the number of sites examined. PPD and CAL were recorded at six points around each tooth except third molars. Frequency of daily brushing of teeth, use of other oral hygiene aids and number of missing teeth due to periodontitis were also recorded. Presence or absence of bone loss was determined by radiographs using long cone technique for all the participants. Diagnostic criteria and clinical case definition for periodontitis were based on the consensus report of workgroup 2 of the 2017 World Workshop on the Classification of Periodontal and Peri-Implant Diseases and Conditions (23). Further, all the subjects were sub-categorized as having stage I, II, III or IV periodontitis based on the staging proposed by Tonetti *et al*. (24).

Calibration trials were done before the commencement of the study. Determination of intra-examiner reproducibility was done by examining randomly selected quadrant in ten patients who were not part of the study. The re-examination was done twice at the same visit by the examiner and intra-class correlation coefficients for mean PPD and mean CAL were 0.943 and 0.929 respectively. The inter-examination difference was within 1mm in 94.5 % of PPD and 92.8% of CAL measurements.

-Statistical analysis

Statistical analysis of the collected data was done using SPSS version 20.0 (IBM Corporation, New York, NY). Descriptive statistics – mean and standard deviation for continuous variables and counts (n) and percentages (%) for categorical variables were calculated. The normalcy of the data was checked using the Shapiro- Wilk test. Data that did not follow normal distribution were calculated and compared between the two groups using Mann-Whitney U-test while data that exhibited normalcy were compared using unpaired t-test. Fischer’s exact test was used to compare the frequency of periodontitis among the two groups. Logistic regression analysis was performed to correlate between the demographic data and stages of periodontitis. Odds ratio (OR) was calculated at 95% confidence interval (CI). A *p-value* <0.05 was considered significant.

## Results

Table 1 shows the data comprising of participant characteristics – age, gender and BMI and frequency of daily tooth brushing and use of other oral hygiene aids of PsA and control groups and rheumatological parameters – ESR and CRP values of PsA group. No statistically significant difference was found between the groups concerning BMI, frequency of daily tooth brushing and use of other oral hygiene aids.

**Table 1:**
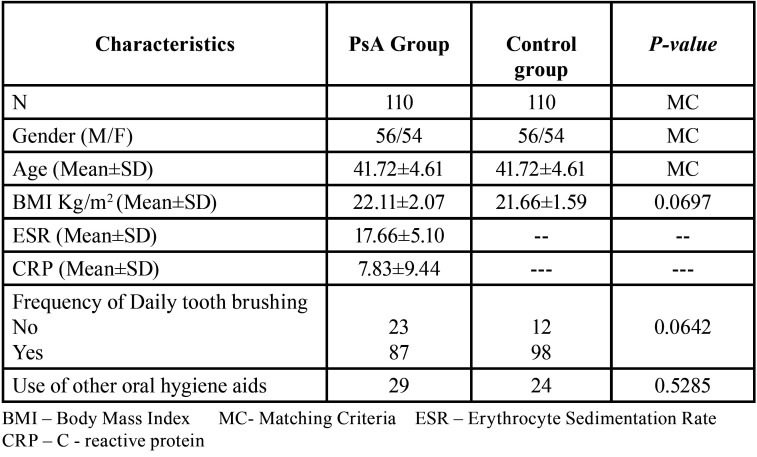
Participant Characteristics.

The percentage distribution of periodontitis in PsA and control group is presented in Table 2. The frequency of periodontitis (58.18% vs 42.72%, *p* = 0.04) was found to be significantly higher in the PsA group as compared to the control group. Also, 48.43% of patients with PsA had stage III periodontitis which was significantly higher as compared to the ones found in systemically healthy controls (27.08%) (*p* = 0.0312). However, when the frequency of stage I and stage II periodontitis was compared between the two groups, no statistical difference was found that reached significance. Both the groups exhibited a generalized pattern of periodontal destruction as compared to the localized and molar-incisor pattern, with more number of PsA patients demonstrating a generalized model in all the stages of periodontitis as compared to systemically healthy compatriots with periodontitis (75% vs 66.66% stage I, 72% vs 65% stage II, 70.96% vs 69.23% stage III periodontitis). Molar- incisor pattern was found only in stage II (3.45%) and stage III periodontitis (6.45%) patients with PsA.

**Table 2:**
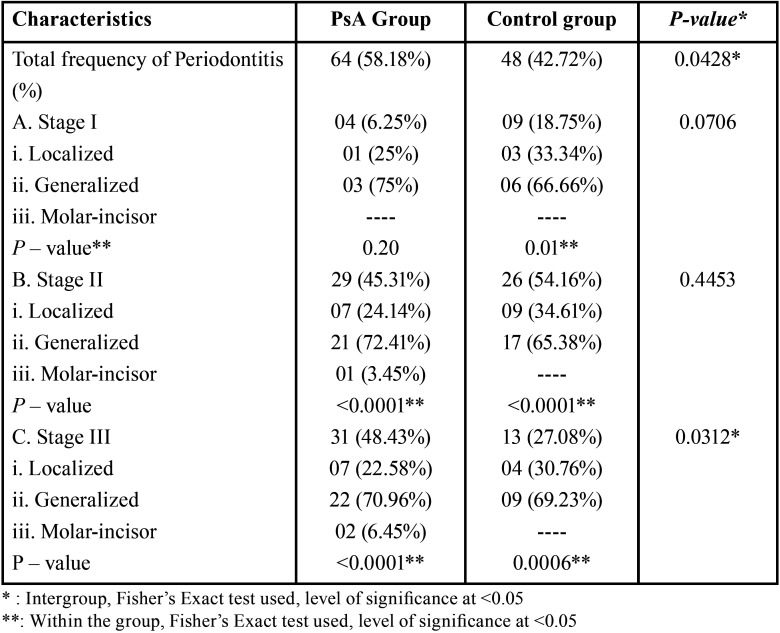
Prevalence of Periodontitis.

Table 3 depicts the comparison of periodontal parameters between the PsA and control group. No statistically significant difference in mean PI score was observed between the two groups. However, the total mean values of BOP (0.57 vs 0.43, *p* = 0.009), PPD (4.23 vs 3.62, *p* = 0.0001) and CAL (3.04 vs 2.53, *p* = 0.006) were found to be significantly higher in PsA group as compared to the control group. When the mean scores of BOP (0.87 vs 0.75, *p* = 0.002), mGI (1.80 vs 1.52, *p* = 0.03), PPD (5.06 vs 4.55, *p* = 0.001) and CAL (3.48 vs 3.13, *p* = 0.007) were compared among periodontitis patients in both the groups, the results reached statistical significance with reportedly higher values in PsA group. Similarly, statistically significant results were obtained among stage II and stage III periodontitis patients when mean values of BOP (0.84 vs 0.72, *p* = 0.04 for stage II; 0.92 vs 0.80, *p* = 0.03 for stage III), mGI (1.87 vs 1.54, *p* = 0.02 for stage II; 2.12 vs 1.6, *p* = 0.01 for stage III), PPD (4.83 vs 4.25, *p* = 0.0007 for stage II; 6.81 vs 5.98, *p* = 0.02 for stage III) and CAL (3.44 vs 3.08’ *p* = 0.02 for stage II; 5.62 vs 5.05, *p* = 0.03 for stage III) were compared between PsA and systemically healthy control group.

**Table 3:**
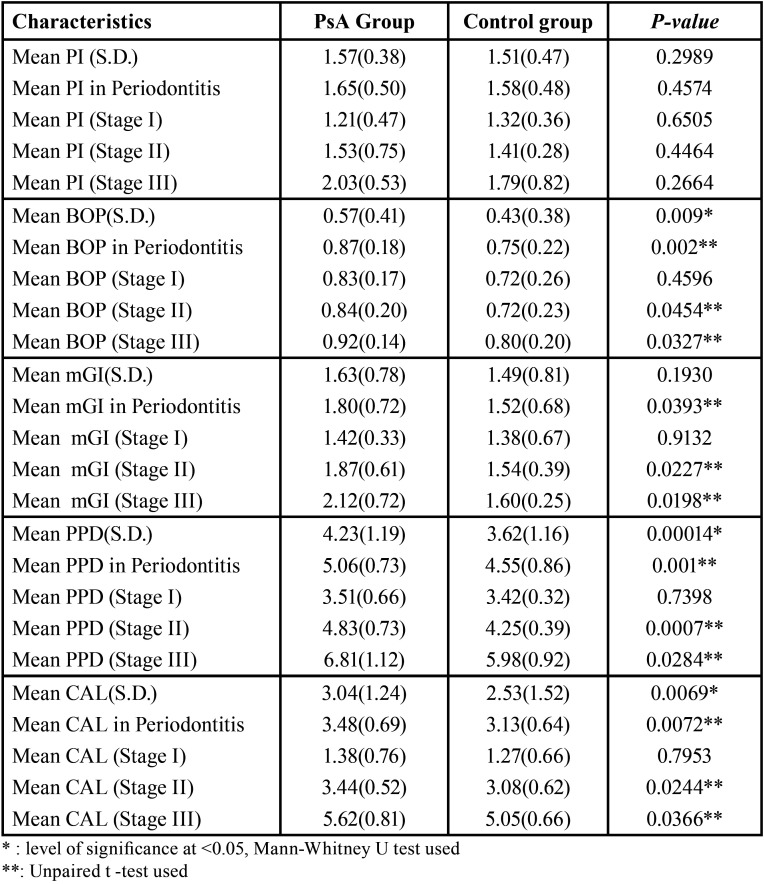
Periodontal Characteristics.

Table 4 shows the magnitude of the association of PsA status and demographic variables against the odds of having periodontitis. The results revealed that the odds of having periodontitis (OR: 1.7971, *p* = 0.03), especially stage III periodontitis (OR: 3.3177, *p* = 0.001) was significantly increased in patients with PsA. Among age, gender, BMI, daily tooth brushing habit and use of other oral hygiene aids, only age remained a significant predictor of having periodontitis (OR: 1.1032, *p* = 0.001), stage III periodontitis (OR: 1.1813, *p* = 0.002) in particular.

**Table 4:**
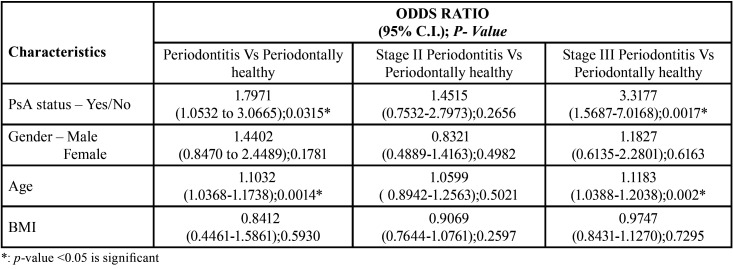
Logistic Regression Analysis.

Additionally, after controlling for age, the odds of having periodontitis (OR: 1.8818, 95% CI – 1.0075 – 3.5148, *p* = 0.04), stage III periodontitis (OR: 3.4113, 95% CI – 2.3182 – 5.0198, *p* < 0.0001) in particular, among patients with PsA, was found to be significantly higher as compared to systemically healthy controls while the difference between crude OR (1.7971 for periodontitis, 3.3177 for stage III periodontitis) and adjusted OR (1.8818 for periodontitis, 3.4113 for stage III periodontitis) was less than 10%. The model fit test showed adequate fit with a sensitivity of 89.2% and specificity 89.6%. The correlation coefficient of the model was 0.715 and it was found to be significant (*p* = 0.001).

## Discussion

Age, gender, BMI, smoking status and presence of certain systemic diseases like diabetes mellitus or cardiovascular diseases are some of the variables that can act as potential confounders and affect the data interpretation. In the present study both the groups under study were age and gender-matched. BMI values were comparable between the two groups and did not attain significance. Also, daily tooth brushing habit and use of other oral hygiene aids did not reach statistical significance. Since ample amount of literature has demonstrated the effects of tobacco usage on periodontitis-systemic disease associations25,26, participants with a habit of consuming tobacco in any form were excluded from the study. To avoid misinterpretation of data as periodontitis is also linked with several co-morbidities like diabetes mellitus (27), cardiovascular disease (28), respiratory diseases (29), adverse pregnancy outcomes (30); etc patients who had systemic diseases other than PsA were excluded from the study.

The major findings of the current study revealed a significantly higher frequency of periodontitis in PsA patients than in systemically healthy controls. Also, patients suffering from PsA had more severe periodontitis (generalized in extent), as compared to the healthy control group which has been described in terms of significantly higher frequency of stage III periodontitis and higher mean values of PPD and CAL in such patients. While PPD is the measure of the current disease activity, CAL is the measure of past disease activity and considered as an important criterion for assessing the severity of periodontitis (31). Moreover, logistic regression model also showed that patients with PsA had higher odds of developing periodontitis as compared to systemically healthy individuals. PsA patients were three times more likely to develop stage III periodontitis as compared to systemically healthy controls. Hence, these findings suggest a possible association between the two diseases under study.

The findings of the present study were in line with the study done by Ancuta *et al*. where around one-third of the PsA patients had moderate to severe periodontitis (10). Similarly, the results of the study done by Sezer *et al*. revealed significantly higher mean PPD and mean CAL in PsA patients with periodontitis as compared to PsA patients with healthy periodontium (32). Moreover, the risk of periodontitis patients and incidence was found to be higher in PsA patients in a Taiwanese general population (33). Similar results were reported by Egeberg *et al*. in a nationwide study in the Danish population that the patients with PsA were at highest risk of developing periodontitis (34). In the study done by Ustun *et al*., the percentage of PsA patients having periodontitis did not attain statistical significance as compared to systemically healthy individuals (35). Also, Ustun *et al*. reported that patients with PsA did not exhibit a significantly higher PPD, and increased severity of periodontitis in the PsA group was due to significantly increased CAL (35). These results were not in agreement with that of the present study. This difference could be due to the inclusion of patients who were under long term use of medications, like NSAIDs, corticosteroids, DMARDs and TNF-α inhibitors, which appear to have protective effects against periodontal destruction (36-38), while in the current study only newly diagnosed PsA patients not under any treatment for the disease were included. Moreover, in the present study, despite similar PI values and plaque control methods in both the groups, higher inflammatory activity as reported by significantly greater gingival bleeding and modified gingival index score was noted in patients with PsA and PsA patients with periodontitis particularly, patients with stage II and stage III periodontitis as compared to systemically healthy compatriots, suggesting a possible reason behind the increased periodontal destruction in such patients. The difference might also be a result of impaired host response in such patients indicating greater susceptibility to periodontal destruction.

Also, the findings of the current study showed that a significant relationship between age and periodontitis exists, giving a possible age-dependent effect. However, after controlling for age, the association between PsA status and periodontitis remained significant with thrice the odds of having stage III periodontitis in such patients as compared to systemically healthy individuals. For the question of whether or not age confounds the PsA status and periodontitis association, the results exhibited limited evidence of confounding since the difference between the crude OR and adjusted OR was less than 10%.

Not much is known about the pathophysiological link between periodontitis and PsA. It is hypothesized that mitochondrial dysfunction, angiogenesis and onset of oxidative stress by increased production of reactive oxygen species (ROS) seem to be present since the onset of early disease and might be considered as important processes associated with the pathophysiological association of periodontitis and PsA (39). The crosstalk between the innate and adaptive immunity that sustain chronic inflammation, resulting in dysregulation and excessive production of various pro-inflammatory cytokines like TNF-α, IL-17, IL-1β, IL-22 and IL-2316, (40,41) suggesting a possible link between PsA and periodontitis. For instance, IL-17 has been reported to possess osteoclastogenic properties that stimulate the expression of the RANKL, which in turn results in osteoclasts’ activation and maturation (42). The mature osteoclasts, being bone-resorbing cells secrete lysosomal enzymes that degrade the bone matrix (40). Furthermore, evidence also suggests that the pro-inflammatory lymphocytic invasion in PsA cases is not only localized to skin or joints but have also been found in blood cell isolates confirming the presence of systemic inflammation in such patients (43). A previous study was done by Moen *et al*., also confirmed the presence of an increased number of periodontal pathogens in the sera and synovial fluids of PsA patients compared to controls suggesting a possible role of periodontopathogens in the initiation and perpetuation of joint inflammation in PsA (15).

The limitations of the present study include its case-control design with a limited sample size so that the estimation of temporal frequency of development of periodontitis in PsA patients gets arduous. Also, association between different clinical patterns/disease activities of PsA with periodontitis could not be made in the current study; further research assessing the same is encouraged. Studies with different experimental designs incorporating biochemical, immunological and various other molecular analyses are required for getting a better understanding of the relationship between periodontitis and PsA.

## Conclusions

Within the limitations of the study, it can be concluded that a possible relationship exists between patients suffering from PsA and severity of periodontitis in terms of PPD and CAL. Although this requires further investigations, a multidisciplinary approach is warranted in patients with PsA. The dental and medical health professionals should be aware of this relationship depending on which, they should carry out adequate treatment strategies involving periodic periodontal evaluation and care.
